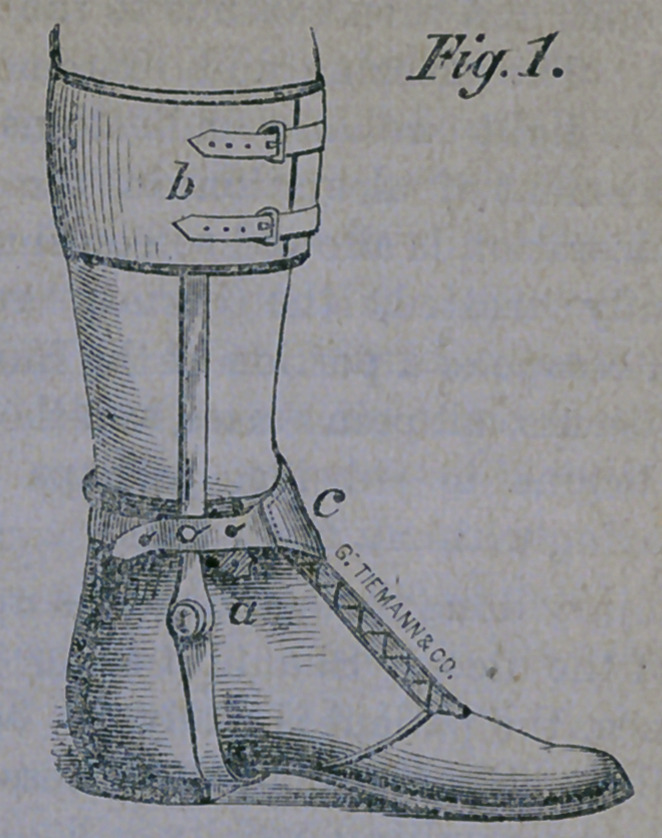# Club Foot

**Published:** 1873-10

**Authors:** 


### CLUB FOOT.

The practice of orthopædic surgery has become so popular of late years, and such good results have been obtained by those skilled in this branch of surgery, that many incompetent persons have attempted to "straighten" deformed feet and limbs, only to increase the difficulty they attempted to remove, and in some instances rendering limbs that were before useful, entirely helpless. Indiscriminate cutting, in club foot, has been productive of much failure and considerable maiming of the patient. The competent surgeon will always be able to judge of the *cause* that produces the deformity, and will vary his treatment to suit the exigencies of the case.

The most common form of club foot is that in which one or both feet turn in so that the child stands upon the outer edge of the foot, the sole turning up, in the direction of the opposite leg. This species is known to surgeons as *talipes varus*, and is aptly illustrated in the accompanying cut:

It is usually caused by an undue contraction of the flexor muscles, upon the side of the deformity, or a paralysis of those upon the side opposite to which the foot is turned. Should the large tendon of the heel (*tendo achillis*) be greatly contracted, the heel will be drawn up, so increasing the deformity.

It must be manifest to almost any observer of ordinary intelligence, that the treatment for the deformity cannot be identical whether it depends upon paralysis of a set of muscles, or undue contraction of another set. Although the deviation of the foot from its natural position, will appear precisely the same to the eye of all, save the surgeon, yet the treatment must widely differ. To cut the contending muscles that unite in mis-placing the foot, when such malposition depends upon paralysis of the muscles upon the opposite side, would leave the foot dangling like a flail upon the end of a stick, rendering the limb entirely useless. Such a condition of affairs requires a nicely adjusted apparatus, similar to the one illustrated in our cut, where the paralysed muscles are supplied artificially, contending with the healthy and contracting ones, restoring the foot to its normal position, while suitable treatment can be adopted for restoring the lost power of the faulty muscles.

When the shoe is fitted and applied, the spiral spring *a* draws the the toes outward, the shoe being jointed at *c* so as to swing in the direction desired. A stout, steel, vertical bar is firmly rivited to the sole of the shoe, and by means of a well padded steel band, is buckled to the leg at *d*. A spiral spring is also fastened to this band, runs under the pully *b* and is fastened near the toe of the shoe. This arrangement overcomes the contraction of the large, heel-tendon, and brings the heel down to a level with the sole of the foot. A strap is buckled across the instep, depressing and securing the foot within the shoe, and with a joint, corresponding to the ankle-joint, the apparatus is complete and answers all requirements.

This ingenious contrivance is the invention of Messrs. Geo. Tiemann & Co., the celebrated surgical instrument makers of New York, and is the one which we are constantly in the habit of applying in this form of club-foot, and to which we attribute so much of our success.

But should the deformity depend upon veritable and abnormal contraction of the muscles, the instrument referred to will require the aid of surgical interference, when the heel-tendon, and perhaps those of the sole of the foot, will be required to be severed before the difficulty can be rectified. It will at once be seen therefore, that such cases should not be entrusted to traveling doctors or inexperienced surgeons. Under proper and skillful treatment, in the hands of an experienced surgeon, nearly if not quite all of these deformities of the feet can be completely rectified.

After the tendons have been cut, and the proper apparatus worn until the foot is rendered straight, a supplementary shoe is then worn, to give support to the ankle and assist in keeping the foot straight until the parts have become accustomed to their new position. Such a shoe is represented below, with an elastic band running from the toe, to overcome the power of the great tendon at the heel, and keep that portion of the foot in its normal position. This apparatus can also be used as a night shoe, to keep up the steady stretching of this tendon, while the patient is sleeping.

*Fig. 1.*

By means of the apparatus here explained and illustrated, any case of *talipes varus* or inturning feet, of a child, can be effectually straightened. In all instances where the patient is in ordinary good health, this means of restoring the feet should be early applied,—before the child is permitted to stand upon its feet, for then the bones are soft, the tendons tender, so that very little or no surgery is necessary to effect the cure.

Other forms of club foot exist, all of which are amenable to the same means of cure, the apparatus being varied to suit the condition of the case.

We hope we have clearly illustrated to parents the means which are adopted by orthopædic surgeons now-a-days, to rectify the deformities of children's feet, and that having an understanding of the matter, and the requirements to be fullfilled, they will not hesitate to place their little ones under the care of suitable surgeons, before such deformities are rendered more difficult of cure, by means of their procrastination.

Extra Edition.—We send out a large number of extra copies of the Bistoury this quarter, with the hope of securing subscribers among those who have never chanced to see our journal. Our subscription price is so low, and the information contained in every issue of the journal so great, that we expect to find no difficulty in extending our circulation.

Plain Speaking.—It would be more obliging to say plainly, we cannot do what is desired, than to amuse people with false words, which often put them upon false measures.

## Figures and Tables

**Figure f1:**
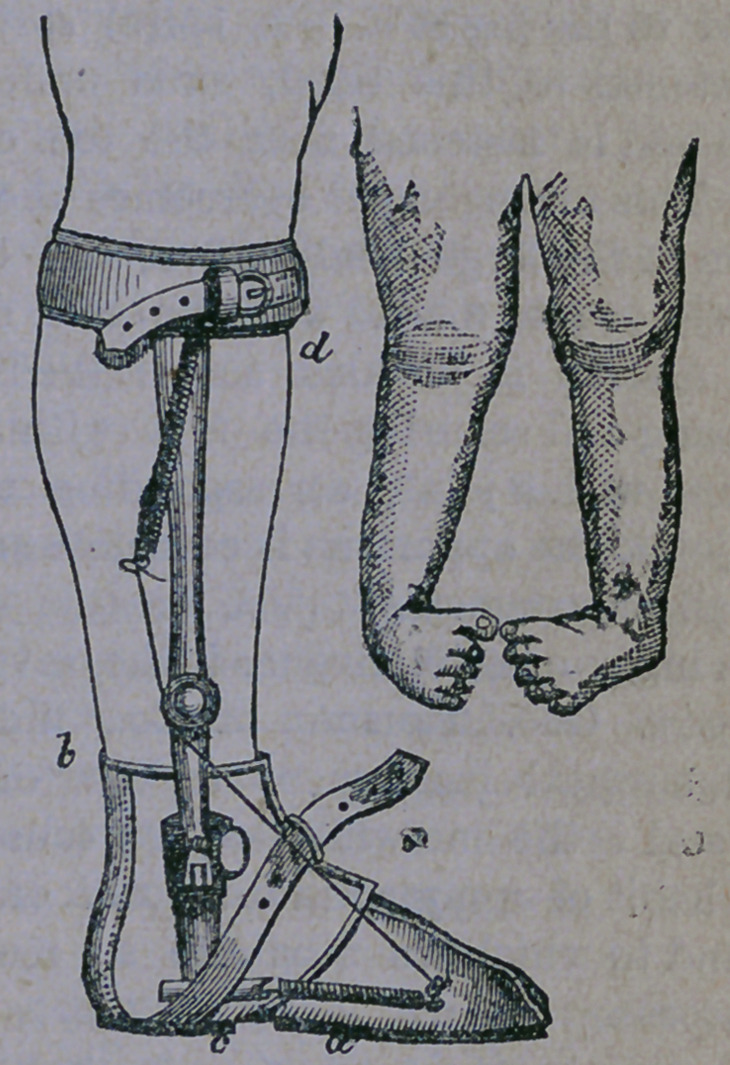


**Fig. 1. f2:**